# Supplementary material for: Patterns of Intron Gain and Loss in Fungi
Source: PLoS Biol. 2004 Nov 30;2(12):e422. doi: 10.1371/journal.pbio.0020422 (PMC532390; doi:10.1371/journal.pbio.0020422)
Supplement: Table S1 — Also available at http://genes.mit.edu/NielsenEtAl/. (4.3 MB ZIP). [file pbio.0020422.st001.zip › NielsenEtAl/html/463.html]

AN4623.1.NCU02349.1.MG03510.1.FG07940.1


```
 CLUSTAL W (1.82) Multiple Sequence Alignments - Introns Inserted


Sequence 1: MG03510.1	1508 aa
Sequence 2: FG07940.1	1463 aa
Sequence 3: NCU02349.1	1500 aa
Sequence 4: AN4623.1	1483 aa
Alignment Length: 1521 aa
Number Identitical Residues: 566 aa
Alignment Score (without introns) 30041


MG03510.1 	MIYLSRLPWGLRVSLLAALAQLQFVPLAAGAPSVEVSMQAAFPSPPYLLELL2ETAAQEN
NCU02349.1	MIRLSQLPWGLAAAIVTATWLPSHH--VAASPSVDVALKAAFPSPPYLVELL2ETAASEN
FG07940.1 	---------MLRLSQYMAMAVLAAT-AASASPSVNVGMHAAFPHGPYLLELL2ETAAGEN
AN4623.1  	MLLRSGIVASWRLSCVVIATLLTAR--ATSSPSVNVALQASFDSGPYLIELL2ETAAEEN
          	    :        :             .:.:***:*.::*:*   ***:*** **** **

MG03510.1 	DTAYFPLLDRIASGHFAKAGTDRELYEKFLQVLQEDGHITSPDALSTFKLALSMRSAAPR
NCU02349.1	ATAYFPLLDRIAKGDFAQAKSDKELYEKFLQVLQEDGHVNA-EALSTFKLALSLRSAAPR
FG07940.1 	STAYFPLLDKIASGHFASANSDAELYHQFLQVLQEDRHIIARDALSTFKLSLSLRAAAPR
AN4623.1  	ATSYFPLLDRIAEGAFDDYLTDKELFDRFLQVVVEDGHLSSPESVSSFKLSLSIRSASPR
          	 *:******:**.* * .  :* **:.:****: ** *: : :::*:***:**:*:*:**

MG03510.1 	IEAHYQHYRTAVEPKFQGK-ADSCPEWILKSGEIFCSVDETKGVANIVGDA~LSKDIPFD
NCU02349.1	VEAHYQYYTTAVEPYVVAD-EDGCDLWFLFNGKQYCKASLQASSDHVNSNS~RERTLPFD
FG07940.1 	IEAHYQYYSTAVDPESQIDGAGNCQSWALIDNQKYCSPDLDVAVEGKVVSK2QAKVLPFD
AN4623.1  	IAAHYQFYNTSVQHSLMAAQDAACPVWVHSDGKQYCSSAMERAQQDVEGDL2DGRALPFD
          	: ****.* *:*:          *  *   ..: :*.     .      .    : :***

MG03510.1 	HQLGDGS-EGVILYADITSPGFGAFHDTLATAARNGGPSYRLRHRSITIASKQPLYLSGY
NCU02349.1	RQFGSGH-QEIVLYADITSPAFGKYHEAAMELARKGEASYRLRYKRSLGQSDDALAVNGY
FG07940.1 	RVLGIG--KDAILYADPTHASFGPFHDVLSKAARQGDVSYRLRYRRSPGVSNTPLPVSGY
AN4623.1  	RILGPNSLPPVILYADVASPMFKDFHQTLSALAKEGEISYRVRYRPPQHWISRPLFVSGY
          	: :* .     :**** : . *  :*:.    *::*  ***:*::      . .* :.**

MG03510.1 	GVELALKRTDYIVIDDRANEKTTQDDSTQKPLVGGSSEIVLNGDEEVADLKPLSASELEE
NCU02349.1	GVELTLKRTDYIVIDDRDTGGDKATGDDSQKTIRSDSELVLDEDEEVADIKPLEKSELSP
FG07940.1 	GVKLDLKRTDYIVIDDRE-----ATQETQKPHI--VADVDLDTDEEVADLKPLSSSELAS
AN4623.1  	GVELALKRTDYIVIDDRD-----AEQRGP------NSIESAGTEEAPDDLKPLSSSEVAR
          	**:* ************                   :    . :*   *:***. **:  

MG03510.1 	LGLKAADFILRGDRPFETLLKLTQDFPKYSASVSAHNVSTGFRTEHSANRMLLQIPSGSN
NCU02349.1	LAVKAASFVMQNESPFDTLLKLTQDFPKYSTKLGKHNVSKEFLAEHEYN-RQLLVPPGAN
FG07940.1 	LGLKTASFILKSDNPMDALLKSTQDFPKFSASIASHEVTPGFAQEQEKN-VAAGVPSGIN
AN4623.1  	LGLNTVSYVANSEDPLNTLVKLSQDFPKYSATIAAYNASTQLHKEIKAN-RLGMLPPGAS
          	*.:::..:: ..: *:::*:* :*****:*:.:. ::.:  :  * . *     :*.* .

MG03510.1 	ILWMNGVQLIERQIQSFGLVDVLRRERQLINEARSLGLSGQEAVDLLSHSDVASSKASDE
NCU02349.1	VLWMNGVQMVDRQVQPFGLVDMLRRERKLINGALDLGLTGQQAVSLLGHDEVAAAKATEE
FG07940.1 	FLWMNGVQLIERQIEPFTLIEMIRRERKLIDGVREIGFNGQQAVSLLGHSEIASSKADDE
AN4623.1  	AIWINGIQIDPRQIDAFFLVDHLRRERKLIDSFRSLGLSAKQAVDLMTHETVTEATAQGT
          	 :*:**:*:  **::.* *:: :****:**:   .:*:..::**.*: *. :: :.*   

MG03510.1 	-PARFDWRDEKEDGKVIVWLNNLEKDKRYENYSPHIMT~LLSGGIPGQLPPIRRNIFNIV
NCU02349.1	EPRRFDWRDEPEGGQVIIWLNNIEKDKRYAEYSPSVWA~LIQHFGQG-LPQVRKNLFNLV
FG07940.1 	-PPRFDWTDRLEDGKAVMWLNDLETDSRYQKFPSDLTA0LLQRAYPGQLPQVALNLFHVV
AN4623.1  	-PQRYDYRDESEGGGVIIWLNDLEKDARYESWPNQLTA0FLQRTFPGQLPAVRRDLNNIV
          	 * *:*: *. *.* .::***::*.* ** .:.  : : ::.    *.** :  :: ::*

MG03510.1 	LPVDFSRASDMALATSQLIGFVKQRIPIRFGLVPLATTTEAEDQAKVVYHLLQTYGLSSL
NCU02349.1	VPVDFSRAEDVTLVTRQLLAFMKRGIPVRFGLVPLTPTGEAVEQAKVLYHLLNTYGLAAM
FG07940.1 	APIDFTDLED-GRAFGQLTQFMQRGITIRFGIVPLATTPASIAQAKVVYHLMETYGFESL
AN4623.1  	FPVDLTRMDDVDLIVSTIQMFVKRKIPVRFGIVPLATSEGSIAQLKVAHYLQETFGLASL
          	 *:*::  .*       :  *::: *.:***:***:.:  :  * ** ::* :*:*: ::

MG03510.1 	MAYLEL~CLDGSTTG-PNQEAFDAAIKDRTVRSELTALSLKDVLASEAYTNQVTLAKQWS
NCU02349.1	STYLEK~SLEASSTDKPNEDIFSLATKDREIRPDHEALPFKHISASEELEKQVHRAKHWC
FG07940.1 	ITYLQE~SEEGPEGA-ANKRSFAKAIDGREPMPAMTKMTLSEVLEAKSYAQKVKAGQAWA
AN4623.1  	MTYLEQ0GLKRNKLAVPDKTCFLKATEDRTPRGEKQPLSLDDILTNEGLDTSVSRTGQYQ
          	 :**:    .      .::  *  * ..*        :.:..:   :    .*     : 

MG03510.1 	SRLGAHSAVPPLFVNGFALPRDSENRWMGMMSGRISGDLRSLQEGIYYGTINDETNVPEI
NCU02349.1	ERLRADTDIPPAFINGFAIPR--EEDWLRNMNHKLMVDLQMLQQAVYYNKVNDHTNVPAF
FG07940.1 	SRLNAATPVRPILVNGMVIPR--EKNWVQVMGQRLTEDQQTIQKAVYFGHVNEDTPVSDL
AN4623.1  	NRLGIKAGDSLIFVNGIPVMR--GENWPQEMSAKIERDLRLVQQGIAEGLFEEDAWLPSF
          	.**   :     ::**: : *   : *   *. ::  * : :*:.:  . .::.: :. :

MG03510.1 	FLADASSRRNHYIFPENDNGLTILDVNKVYTDHDDLFTKAAVIESSPDFDREAWALLTVF
NCU02349.1	FLENAIARRNTFIYPEDANAVKVLNVNKVYSEHQRLFSKVPVVEADDTAPKEDWAVLTVV
FG07940.1 	FLKTALSKRNAHIFPDDDKTLRILDVNKLYTDHAELFSKIAVLPADVESAKEDWAVLTVI
AN4623.1  	FLSQTFGRRVPWIVPEDPKDIELVDLVKVAAQLGALLDKVPRVAAHVEPLES--VHVVVV
          	**  : .:*   * *:: : : :::: *: ::   *: * . : :     ..  . :.*.

MG03510.1 	VDLDSKDGREILLSALRFRRSNPGVRLEIVHN-AVKKTSHDIN-SNLKEQSDRLLAAESD
NCU02349.1	TDLNSVEGQKLLYFALRFRQEHQGVRLDIVHNPADLANSPSIMNQRVKAKESSLLEVTRL
FG07940.1 	ADLNTNDGQDLLLTALKFKRNNQGIRLDLVHNPSLPSNAHAIN-GAFKLSEVKLVEMRCK
AN4623.1  	GDFGTRAGLKMLISTLEMQLKQDKTEILLVHNPEDPLTNAAAS-SLIYRSLKEGNGVDST
          	 *:.:  * .:*  :*.:: .:   .: :***.    .        .  .          

MG03510.1 	EGLVAILEEIRHAPQTNIGLEYAQALERFQRAAHFEPGTSILMLNGRVVGPIQSASEFSE
NCU02349.1	VDLETILEEGKPEADP----DFDADLASFLSGFNLKSGDNMLILNGRIVGPIASANDFLK
FG07940.1 	DDLKAVLEAPWTAEED----GFGTALANFLLTSNIQPGTKGLLLNGRFVGPLPSSVSFED
AN4623.1  	RILADITSSTSSDVDAQEASEFWLKFQPLVEALGLSSGMNGIIVNGRVIKPSQ---DVTA
          	  *  : .      : . .  :   :  :     :..* . :::***.: *     ..  

MG03510.1 	SDFQTFLEFEQNSRIIPVYKALEELGLGDRLSDPVAAAKLTSITALSTISDLPDGAFENP
NCU02349.1	EDFAEFLRTERMNRILPVYKAIEDLGLTDKVSGPLAAAKLTSVTALSGISDTPQGIFDSA
FG07940.1 	DDLKLLLEFEQRSRILPVYAAIKELGFADRLSDPISAAKLTSITALSTISDLPQGIFESA
AN4623.1  	EDLGQLLLYEDINRIGPVTKAAKDLGLGSRIPDPLSFAKLTSLMAISTVSDVPEGLYETS
          	.*:  :*  *  .** **  * ::**: .::..*:: *****: *:* :** *:* ::..

MG03510.1 	PTLRISSFKLWNSTHTGFEVGDPSKASIFFTAVIDPASELGQKWTPLLKVLSELEGVHLK
NCU02349.1	PPIRTTAYNRLNTTYTSFHVGNPETATIFFVAVINPASEMGQKWAPILKVLSELEGVHLQ
FG07940.1 	PSIRSTLYNTWNATHSTIEVGNPETASVHIAGLLNPTSEQGQRWAPILKVLSELDGIYLK
AN4623.1  	SDVRVKFFEEWNESSSVITVPGPEDPVITISASLDPTSEMAQRWLPILKVISDLDGVRLR
          	. :* . ::  * : : : * .*. . : : . ::*:** .*:* *:***:*:*:*: *:

MG03510.1 	VFMNPRDRLEELPVKRFYRFVMDSAPVFDEAGKLEVPSASFKGLPSEALLNLGMDVPPAW
NCU02349.1	VFVNPQTELSELPVKRFYRYVLESAPSFDESGKVKALSATFNGVPPETLLVAGMDVPPAW
FG07940.1 	LFMNPKELTGELPIKRFFRYVLDSTPSFDDSGHVQSPKATFKGLPSEALLTAGMDVPPAW
AN4623.1  	LFMNPRDELRELPIKRFYRYVLDAQPSFSQDGSVSRPTATFSGVPVEALLTLGMDVPPYW
          	:*:**:    ***:***:*:*::: * *.: * :.  .*:*.*:* *:**  ****** *

MG03510.1 	LVAPKVSVHDLDNIKLSSVTSD-----VTAIYELENILIEGHARSTDSGTP-RGVQLVLG
NCU02349.1	LVASKVSVDDLDNLRLKDIKAKRNTEHVEAIYELENILIEGHSREFPSGSPPRGVQLVLA
FG07940.1 	LVAAKDSIQDLDNIKLSSIKSD-----IDVVYELENILVEGHSRDGKRGAP-RGAQLALA
AN4623.1  	LVAPKESIHDLDNIKLSSVKGGSD---VDAIYALEHILIEGHSRDITTKSPPRGVQLVLG
          	***.* *:.****::*..:..  .   : .:* **:**:***:*.    :*.**.**.*.

MG03510.1 	TESNPRVADTIIMANLGYLQFKANPGIYDITLEKGRSSDIFYVESIGAHGFSPVAGDETT
NCU02349.1	TEKHPHFADTIIMANLGYFQFKANPGMYSIHLMEGRSSDIFTLDSVGAQGWSPVPGDETT
FG07940.1 	TEKDPLITDTIVMANLGYFQFKANPGFYSIRLKEGRSSEIFTIESAGAHGYAAVPGDEGT
AN4623.1  	TEDNPYFADTIVMANLGYFQFKAQPGLWTINLKPGRSEQIFNLESLGRLGYSPQAGDDSN
          	**..* .:***:******:****:**:: * *  ***.:** ::* *  *::. .**: .

MG03510.1 	EVALLDFLGTTLYPRLRRNPGKENLDVLEESGAAPSGNAGGAMDFVNKGLKFAESLLGGA
NCU02349.1	EVALLSFQGATLYPRLTRKPGMEREDVLQDETTPQDES------LVSKGLKFAEGLFGSK
FG07940.1 	EIALMDFKGTTLYPRLNRKSGMEEADVLES---VDSDNDG----IVAKGLKFAESLLGGA
AN4623.1  	EVALLSFQGKTLFPRLSRKRGYEDEDVLESGLKPGSAMD-----FMSKGLNFASNVLSSV
          	*:**:.* * **:*** *: * *  ***:.     .        :: ***:**..::.. 

MG03510.1 	KGKAKEKSPSELQHAEINIFSVASGHLYERMLNIMMVSVMRHTKHTVKFWFIEQFLSPSF
NCU02349.1	K--PTEKSVSETEHAEINIFSVASGHLYERMLSIMILSVMEHTDHSVKFWFIEQFLSPSF
FG07940.1 	K---SPKEISAQEHAEINIFSVASGHLYERMLNIMMVSVMRNTKHSVKFWFIEQFLSPSF
AN4623.1  	G----VTSKSSDPNADINIFSVASGHLYERMLNIMMVSVMKNTKHTVKFWFIEQFLSPSF
          	      .. *   :*:****************.**::***.:*.*:**************

MG03510.1 	KDFIPHMAAEYGFKYEMVTYKWPHWLRQQKEKQREIWGYKILFLDVLFPLTLDKVIFVDA
NCU02349.1	KSFLPHLAAEYGFKYEMVAYKWPHWLRHQSEKQREIWGYKILFLDVLFPLSLDKVIFVDA
FG07940.1 	KEFIPHMAAEYGFKYEMVTYKWPHWLRQQKEKQREIWGYKILFLDVLFPLSLDKVIFVDA
AN4623.1  	KSFLPNLASEYGFSYEMVTYKWPHWLRAQKEKQREIWGYKILFLDVLFPLSLDKVIFVDA
          	*.*:*::*:****.****:******** *.********************:*********

MG03510.1 	DQIVRTDMYDLVQLDLQGAPYGFTPMCDSRTEMEGFRFWKQGYWERYLKGLPYHISALYV
NCU02349.1	DQVVRTDMYDLVSLDLEGAPYGFTPMCDSRTEMEGFRFWKTGYWANYLRGQPYHISALYV
FG07940.1 	DQIVRTDMIDLVNHDLEGAPYGFTPMCDSRTEMEGFRFWKQGYWANYLRGLPYHISALYV
AN4623.1  	DQIVRTDMFDLVSYPLDGAPYGFTPMCDSREEMEGFRFWKQGYWKNFLRGAPYHISALYV
          	**:***** ***.  *:************* ********* *** .:*:* *********

MG03510.1 	VDLRRFRELAAGDRLRQTYHTLSADPNSLSNLDQDLPNHMQFSIPIFSLPQEWLWCETWC
NCU02349.1	VDLRRFRELAAGDRLRQQYHTLSADPNSLANLDQDLPNHMQFQIPIKSLPQEWLWCETWC
FG07940.1 	VDLNRFRQLAAGDRLRQQYHTLSADPNSLSNLDQDLPNNMQFAIPIHSLPQEWLWCETWC
AN4623.1  	VDLNRFRALAAGDRLRGQYQMLSADKNSLSNLDQDLPNHMQHHIPIKSLPQEWLWCETWC
          	***.*** ********  *: **** ***:********:**. *** *************

MG03510.1 	SDDTQPMAKTIDLCNNPMT1KEPKLDRARRQVPEWNVYDAEIAAVDRKRKGLPVSGDAAS
NCU02349.1	SDETLTKARTIDLCNNPMT~KEPKLERARRQVPEWTVYDEEVAALAKRVREQEEKKAGEV
FG07940.1 	SDDSLTKARTIDLCNNPQT~KEPKLDRARRQVPEWTIYDNEIAALDQRRKGV--------
AN4623.1  	SDESLSRARTIDLCNNPQT~KEPKLDRARRQVPEWTEYDDEIAALARRVAAS---EVEVE
          	**:: . *:******** * *****:*********. ** *:**: ::            

MG03510.1 	EQAAKNPKSRTLAVEP-ENTHIVDEL
NCU02349.1	LEGKKVEEQVIVEEKHEEKEHVIDEL
FG07940.1 	--AGKNENTRSRESE--DKAHTKDEL
AN4623.1  	VEQQILADEIEEEKEAAASEWKKDEL
          	 .     .      :   .    ***
```
